# Global spotlight editorial: Advancing treatment planning to promote equity through AI and automation in clinical medical physics

**DOI:** 10.1002/acm2.70640

**Published:** 2026-06-07

**Authors:** Nataliya Kovalchuk, Laurence Court, Jianrong Dai, Stefania Pallotta, Yi Rong

**Affiliations:** ^1^ Radiation Oncology Department Stanford University Stanford California USA; ^2^ Radiation Oncology Department MD Anderson Cancer Center Houston Texas USA; ^3^ Radiation Oncology Department National Cancer Center/Cancer Hospital Beijing China; ^4^ Department of Experimental and Clinical Biomedical Sciences “Mario Serio” University of Florence Florence Italy; ^5^ Medical Physics Unit AOU Careggi Florence Italy; ^6^ Radiation Oncology Department Mayo Clinic Scottsdale Arizona USA

## 1 INTRODUCTION

Journal of Applied Clinical Medical Physics *(JACMP)* is introducing a new editorial series titled Global Spotlight Editorial, aiming to highlight experts from different countries around the world, identified from the pool of top reviewers, authors, or editorial contributors for the journal. In each feature, a critical topic will be determined by the editors, and invited experts will share their professional viewpoints in an interview dialogue format. Invited experts from diverse regions and practice settings will share their professional milestones and contributions to advancing the field of patient care. This series aims to humanize the global medical physics community and inspire collaboration across borders.

The first topic addressed in this editorial is “Advancing treatment planning to promote equity through Artificial Intelligence (AI) and automation.” AI deep‐learning models are being developed and deployed for automated contouring and treatment planning in Radiation Oncology departments globally. However, alongside these technological advances lies a critical global challenge: ensuring that innovation translates into equitable access to high‐quality radiotherapy worldwide.^1^ While high‐resource centers are increasingly adopting AI‐driven solutions, many institutions, particularly in low‐ and middle‐income countries (LMICs) and regions affected by conflict, continue to face significant barriers, including limited infrastructure, workforce shortages, and restricted access to modern technologies.^2^, ^3^ In this context, AI and automation represent not only technological evolution but also a potential pathway to reduce disparities by standardizing workflows, improving efficiency, and supporting workforce capacity.^4^

At the same time, there is a risk that unequal access to advanced tools, data, and expertise could further widen the gap between well‐resourced and under‐resourced settings. The responsible development and implementation of AI in medical physics, therefore, requires careful consideration of scalability, validation, accessibility, and training, ensuring that these innovations are both clinically effective and globally inclusive.^5^

In this Global Spotlight editorial for the JACMP, we invited three internationally recognized medical physicists, Dr. Laurence Court (United States), Dr. Jianrong Dai (China), and Dr. Stefania Pallotta (Italy), to share their perspectives on the evolving role of AI and treatment planning automation in clinical practice. These experts represent diverse healthcare systems and bring complementary expertise in treatment planning, imaging, and clinical implementation of advanced technologies.

## 2 EXPERT VIEWPOINTS


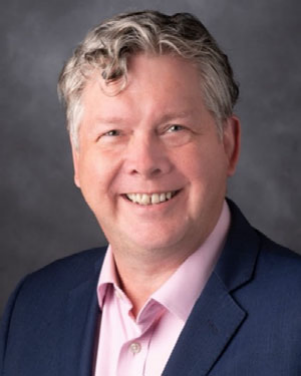


**Dr. Laurence Court** is a Professor in the Department of Radiation Physics within the Division of Radiation Oncology at The University of Texas MD Anderson Cancer Center. He leads the Court Lab, which focuses on the development of the Radiation Planning Assistant (RPA), an automated contouring and treatment planning system designed to provide low‐cost, web‐based radiotherapy solutions, particularly for low‐ and middle‐income countries. His work leverages deep learning and artificial intelligence with a strong emphasis on rigorous clinical validation. Dr. Court's research also includes radiomics, aiming to develop imaging‐based signatures to support clinical decision‐making. His multidisciplinary team includes faculty, trainees, and technical experts. His work is supported by funding from the National Cancer Institute, Wellcome Trust, industry partners, CPRIT, and MD Anderson. He has received multiple awards for excellence in research, education, and scientific publication, including several honors from the Journal of Applied Clinical Medical Physics.

1. *Dr. Court, The Radiation Planning Assistant (RPA). Could you describe the key achievements of this work to date, and what you see as its most meaningful impact on clinical practice and global equity?*

The Radiation Planning Assistant (RPA)^6^ was developed in response to the large and persistent gap in access to high‐quality radiotherapy, particularly in low‐ and middle‐income countries, where workforce and infrastructure limitations make it difficult to deliver standard‐of‐care treatment planning. Over the past decade, it has evolved into an end‐to‐end system for automated contouring and treatment planning, with a focus on reliability and clinical usability. Key achievements include FDA 510(k) clearance, extensive multi‐institutional validation, and early clinical deployment in settings such as South Africa.

Its most meaningful impact is, I hope, still to come as we expand to new countries, helping clinical teams scale their efforts to treat more patients. Our main contribution is not just automation, but the design to deliver that automation in a way that is highly usable, scalable and accessible across a range of settings.

1.1. How does the ARCHERY trial fit into this work?

The ARCHERY trial^7^ represents a natural extension of this work into prospective clinical evaluation across multiple clinics in several LMICs. It is particularly exciting as it is the first large‐scale prospective study of AI‐based radiotherapy planning, with nearly 1,000 patients enrolled. RPA‐generated contours and plans are assessed by independent international reviewers who are blinded to the study site. In addition to evaluating clinical acceptability, the ARCHERY study examines time and cost savings. We are excited that the RPA is providing the AI backbone for this study and are greatly looking forward to the results, with initial findings to be presented as a late‐breaking abstract at ESTRO this year.

2. *As automation and AI‐driven planning systems continue to advance, do you foresee a future where fully automated radiotherapy workflows become the norm? In that scenario, how do you see the role of the medical dosimetrist and physicist evolving or being fundamentally redefined?*

Automation will continue to expand, and more parts of the radiotherapy workflow will become automated, particularly for common disease sites and standard treatment approaches. Fully automated workflows are technically plausible in some settings, but in practice there will still be a need for human oversight, especially for more complex or unusual cases. Variability in anatomy, imaging, and clinical decision‐making means that a completely hands‐off approach is unlikely to be appropriate in all situations. Active engagement of the user (dosimetrist, physicist, oncologist) is vital for the safe implementation of AI. Roles will evolve as tasks such as contouring and planning for standard cases become increasingly automated. There will likely be an increased emphasis on review, validation, and handling of unusual cases.

3. *AI has the potential to both reduce and widen global disparities in cancer care. What do you see as the biggest risk that AI could exacerbate inequities, and what concrete steps are needed to ensure that tools like RPA truly scale in a sustainable and equitable way worldwide?*

AI has the potential to reduce disparities, but the main risk is that it ends up benefiting the same well‐resourced settings that already have access to high‐quality care. This can happen if systems are developed and validated primarily in a narrow set of institutions, rely on infrastructure that is not widely available, or require levels of expertise that are difficult to support in lower‐resource settings. In that case, AI improves efficiency where care is already strong, but does little to expand access.

Avoiding this requires treating equity as a design requirement rather than an afterthought. In practice, this means building and testing systems across multiple sites and populations, including those with different workflows and constraints. It also means keeping the technical requirements realistic, so that tools can function with limited connectivity, staffing, or local expertise. Human oversight remains important, particularly in settings where robustness may vary. Finally, there needs to be a plan for long‐term support, including training, maintenance, and the ability to adapt systems over time. Without that, even well‐designed tools are unlikely to scale in a sustainable way.

4. *Do you want to comment on the work from Dr. Jianrong Dai in China and Dr*. ***Stefania Pallotta*** *in Italy, specifically, where you see commonalities and which aspects of their work may be unique to the social context and patient populations they serve?*

Both Stefania Pallotta and Jianrong Dai share common ground with our work in their focus on improving the quality and consistency of radiotherapy, as well as increasing the role of automation. I am keen to learn from their work. Pallotta's work is largely in an environment where access to radiotherapy is generally available, and the main challenge is ensuring consistent, high‐quality delivery across institutions. As a result, her work focuses on multicenter validation, quality assurance, and reproducibility. Dai's work reflects a different setting, with a stronger emphasis on scale, efficiency, and AI‐driven methods, often developed using large national datasets. Our work with the Radiation Planning Assistant overlaps with both approaches. Like Pallotta, we place a strong emphasis on validation and consistency. Like Dai, we focus on automation and scalability. Their work highlights the importance of validation and efficiency, but also makes clear that additional considerations are needed when applying these approaches across different clinical and resource settings.


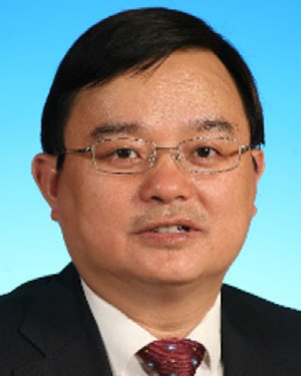


**Dr. Jianrong Dai** is a Professor of Medical Physics at the Chinese Academy of Medical Sciences and Peking Union Medical College, and a Vice Chairman of the department of radiation oncology at the National Cancer Center/Cancer Hospital in Beijing, China.

Dr. Dai's research emphasizes the clinical implementation of emerging technologies, including automation and data‐driven approaches to enhance precision and efficiency in radiation oncology. He has authored numerous peer‐reviewed publications, including the Journal of Applied Clinical Medical Physics, and is actively involved in advancing medical physics practice and education in China and internationally. In 2022, he became a fellow of International Organization for Medical Physics (IOMP), and in 2023, he received the Lifetime Achievement Award from the Asia‐Oceania Federation of Organizations for Medical Physics (AFOMP).

1. *Dr. Dai, you have led several innovative efforts in applying AI to contouring and treatment planning. Which of your contributions do you believe has most challenged conventional approaches, and which do you think has the potential to most fundamentally change the future of radiation oncology?*

My colleagues and I started research on applying AI to contouring targets and organs at risk in 2016^8^ and to treatment planning in 2018.^9^ We have developed more than 33 contouring models, and some of them have been used in our department clinically since 2018. Through these years, the contouring pattern changed dramatically. For example, last year, the targets and organs at risk of 100% of all our patients (11,519 of 11,519) were contoured by an AI model, and then reviewed and modified, if necessary, by physicians. In contrast, the planning pattern has not changed much. AI models are only used to predict initial optimization objectives and constraints for head‐and‐neck cancer patients and lung cancer patients in our department. The usage rate of AI‐base planning was just 13.8% (1584 of 11,519) last year.

Based on our work and similar works done in other centers globally, I think AI‐based contouring has most challenged conventional approaches, including manual contouring and atlas‐based contouring, and AI‐based planning has the potential to most fundamentally change the near future (possibly within five years) in radiation oncology. There is a general shortage of physicists and dosimetrists in low‐ and middle‐income countries and regions. For example, there were only 4172 physicists (including dosimetrists) while there were 1,259,602 patients treated with radiotherapy annually in China mainland according to a national survey performed in 2019. AI‐based planning is expected to play an even more important role in low‐ and middle‐income countries and regions.

2. *AI has the potential to standardize treatment planning across institutions, but could this come at the cost of individualized, patient‐specific decision‐making? How do we balance efficiency and consistency with true personalization of care?*

No. Unlike traditional standardization methods, AI has the potential to standardize treatment planning across institutions and, at the same time, enhance individualized, patient‐specific decision‐making. This is because patient‐specific features can be identified and then be taken into calculations by AI models. How well an AI model can identify patient‐specific features and how well it can take these features into calculations determines its ability to deliver truly personalized care. Although a model that supports personalization better may mean the model has a larger scale and more parameters, and consequently may decrease its efficiency. However, the reduction in efficiency is minor, even negligible, with appropriate hardware acceleration.

3. *AI planning models are often trained on data from high‐resource centers. Do you see a risk that these systems may not generalize well to different patient populations or clinical environments, potentially reinforcing global inequities?*

Yes. We should pay attention to this risk and mitigate it in order to prevent it from reinforcing global inequities. Normally, an AI planning model can only apply to patients who have the same kind of tumor and are going to be treated with the same kind of technology as those patients the model is trained on. That means the model won't be used in a low‐resource center if it does not have the same technology as the high‐resource center where the model is trained. One way to solve this problem is to allow the model to be fine‐tuned with data from the low‐resource center. Another way is to train the model with data from representative centers that are expected to be users of the model. No matter how the model is trained, the model should be accepted and commissioned by medical physicists qualified in AI‐based clinical applications.^10^

4. *Do you want to comment on the work from Dr. Laurence Court in the USA and Dr*. ***Stefania Pallotta*** *in Italy, specifically, where you see commonalities and which aspects of their work may be unique to the social context and patient populations they serve?*

Both Dr. Laurence Court and Dr. Stefania Pallotta have done excellent work on AI's applications in radiotherapy. While the underlying AI methodologies they develop are scientifically universal and not limited by borders, what makes their work stand out is how they translate these technologies to fit specific clinical needs. A good example is the Radiation Planning Assistant led by Dr. Court. While the algorithm itself is generalizable, this project was specifically designed to support radiotherapy practice in low‐ and middle‐income countries.^11^ This project definitely helps alleviate global inequities in access to radiotherapy. And many more such projects are needed, and we as medical physicists should actively contribute in this regard.


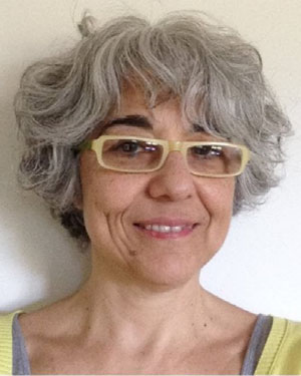


**Dr. Stefania Pallotta** is a Professor of Applied Physics at the University of Florence, head of the Medical Physics Unit at Careggi University Hospital in Florence, and Director of the Specialization School in Medical Physics at the University of Florence, Italy. Her expertise is in advanced imaging, dosimetry, and the application of artificial intelligence in radiation oncology. Her work focuses on integrating AI‐driven approaches into treatment planning and clinical workflows to improve precision, efficiency, and consistency in radiotherapy. Dr. Pallotta has contributed to research in image‐guided and adaptive radiotherapy, with a particular emphasis on leveraging data‐driven methods and automation to enhance treatment planning and decision‐making. She has authored and co‐authored numerous publications, including the ones in the Journal of Applied Clinical Medical Physics. She is actively involved in European collaborations advancing the clinical implementation of AI and emerging technologies. Through her work, she aims to bridge the gap between innovative computational methods and practical, patient‐centered radiotherapy.

1. *Dr. Pallotta, in your 2021 study on the implementation of automated treatment planning in Italy*,^12^ *you found that while nearly half of centers had access to automation, only about one‐third were using it clinically, despite an overall positive attitude among physicists. What do you see as the key barriers that have limited broader adoption, and what needs to change for AI‐driven planning to become a routine standard of care?*

Based on the considerations emerging from the study, the gap between the availability of automated planning and its actual clinical use cannot be attributed to a single cause, but rather to a combination of organizational, technical, and cultural factors. Among the main barriers, economic constraints remain significant for a substantial proportion of Italian centers, delaying both the acquisition and full implementation of these systems. In addition, the time and effort required for system commissioning are non‐negligible, particularly for knowledge‐based approaches; extensive training and validation processes are needed, which may discourage centers with more limited resources or experience. Another important limitation is the lack of adequate training, which can slow adoption and reduce confidence in clinical use. Furthermore, automated planning is more readily integrated into highly standardized environments, whereas its applicability may be more limited in centers characterized by greater clinical complexity and, consequently, lower levels of standardization.

For AI‐based planning to become a routine standard, increased financial investment and broader access to technology are essential to reduce disparities among centers. This must be accompanied by structured and continuous training, enabling professionals to confidently configure and critically evaluate these systems. Equally important is the availability of simpler and more robust tools that are easier to implement. In parallel, greater standardization of clinical workflows, at least for selected indications, can facilitate the integration of automation. Finally, shared validation processes and participation in inter‐institutional audits are key elements for building trust and establishing best practices.

In summary, the transition to routine implementation will depend not only on technological progress but also on the ability of centers to integrate these tools in a gradual, safe, and well‐supported manner, underpinned by appropriate training and organizational frameworks.

2. *Treatment planning has traditionally relied on expert judgment and nuance. As AI systems begin to encode and potentially surpass that expertise, are we at risk of losing critical human insight, or redefining what expertise actually means in medical physics?*

The introduction of AI into treatment planning should not be interpreted as a loss of expertise, but rather as its evolution. It is understandable that concerns may arise regarding a potential reduction in traditional skills, particularly in a field historically grounded in experience, intuition, and expert judgment. As an increasing portion of the process becomes automated, there is a risk that certain operational abilities may diminish, especially when systems are used in a “passive” manner, without genuine engagement in their understanding or configuration.

However, AI does not eliminate expertise; rather, it reshapes its nature. Professional value shifts from the manual construction of plans to the ability to understand, supervise, and manage complex systems. Activities such as validation, optimization, and critical interpretation of results require a high level of knowledge and responsibility. In this sense, the role of the expert evolves from executor to guarantor of quality and of the decision‐making process.

Moreover, automation helps reduce variability and improve consistency, without replacing clinical judgment. On the contrary, it makes it even more central in complex cases, exceptions, and strategic decisions. Rather than a loss of fundamental human insight, this represents a redefinition of the concept of expertise: less focused on manual execution and more oriented toward system understanding, critical thinking, and the informed management of technological innovation. The challenge will be to avoid uncritical use of these tools and to ensure that future generations develop both traditional competencies and new AI‐related skills.

In this context, it is important to remember that no technology is inherently “good” or “bad”: its value depends on how it is used. AI can enhance consistency, efficiency, and quality, but only if it is implemented thoughtfully, with clear oversight of processes and critical decision points.

3. *Looking ahead 10–20 years, do you believe fully autonomous radiotherapy, where imaging, planning, adaptation, and delivery are integrated into a largely self‐driving system, is realistic? What are the biggest obstacles preventing this future?*

Yes, I believe that a highly automated radiotherapy scenario within the next 10–20 years is realistic. The evolution will move toward increasingly autonomous systems, but not ones entirely “left to themselves”: human supervision will remain a key element, especially in the early phases of adoption, to ensure safety, reliability, and clinical acceptance.

The main challenges are not so much related to the ability of AI to generate solutions, but rather to building a robust ecosystem around it. It is essential to integrate highly efficient and qualified control systems capable of continuously and independently verifying each step of the process. This must be accompanied by a rigorous definition of the clinical workflow, with clear identification of steps and responsibilities. Above all, it is crucial to identify critical decision points that must not be bypassed by the machine but preserved as moments of informed human evaluation.

In the longer term, the level of supervision may decrease as confidence in these systems grows, but it is unlikely to disappear entirely, at least until roles and responsibilities are clearly defined. In the near future, a more realistic and desirable model is one in which technological autonomy progressively increases, while the human role becomes increasingly focused on oversight, validation, and the management of exceptions.

4. *Do you want to comment on the work from Dr. Jianrong Dai in China and Dr. Laurence Court in the USA, specifically, where you see commonalities and which aspects of their work may be unique to the social context and patient populations they serve?*

The work of Dr. Jianrong Dai and Dr. Laurence Court shares a strong common foundation: both aim to use AI to make radiotherapy planning more standardized, reproducible, and less operator‐dependent, with the goal of improving treatment quality. This convergence reflects a shared vision that automation can help ensure a more uniform level of care. At the same time, these approaches are shaped by very different social and healthcare contexts. In Dai's case, the work is embedded in a system with large patient volumes, where the main challenge is managing high throughput while maintaining quality and consistency. Here, AI serves to scale expertise and make it more widely available. In contrast, Court's work focuses on settings with limited resources and expertise, where access to radiotherapy is often constrained. In this context, automation is not only about efficiency but also about enabling access to high‐quality care and reducing disparities across patient populations.

These perspectives highlight how AI is not a one‐size‐fits‐all solution, but a tool that adapts to different needs, managing high demand on one hand and expanding access on the other. In both cases, the common denominator remains the use of automation to extend and distribute clinical expertise. Within this framework, our work complements these efforts by focusing on how such technologies can be effectively and safely integrated into routine clinical practice.

## 3 CLOSING REMARKS

As reflected in the perspectives from Drs. Court, Dai, and Pallotta, AI and automation tools are playing critical roles in radiotherapy clinical workflows, particularly for contouring and treatment planning. In high‐income countries, automated contouring has achieved broad clinical adoption and is now routinely integrated into practice, whereas automated treatment planning has encountered more complex problems in clinical deployment and requires more advancement before clinical readiness. This difference highlights a broader challenge. Successful implementation depends not only on technological capability but on thoughtful integration into clinical practice. Importantly, the impact of AI on global oncology remains closely tied to issues of access and equity. While automation has the potential to standardize care and expand capacity, there is also a risk that these advances may disproportionately benefit well‐resourced settings unless deliberate efforts are made to ensure scalability and accessibility across diverse environments. In this context, the role of vendors and the industry is essential. The design of platforms, data requirements, and implementation pathways will directly influence global accessibility. Scalable, robust, and user‐friendly solutions, along with sustainable support and training models, will be critical to ensure that these technologies can be adopted beyond highly resourced centers.

Ultimately, the integration of artificial intelligence into radiotherapy is not about replacing expertise, but about redefining it. As Dr. Curtis Langlotz from Stanford has suggested, it may not be AI that replaces us, but colleagues who know how to use it. In this evolving landscape, the responsibility of the medical physics and radiation oncology community is to ensure that these technologies are implemented thoughtfully, safely, and equitably. The future of radiotherapy will be shaped not only by innovation but by our ability to adapt, collaborate, and extend these advances to improve care for our patients across the world.
